# C-751-06. Pre-existing Mobility Impairments Increase Likelihood of Shelter Discharge After Burn Hospitalization

**DOI:** 10.1093/jbcr/irag033.011

**Published:** 2026-04-06

**Authors:** Maya Matheny, Erin E Ross, Haig A Yenikomshian, Andrew Humbert, Karen J Kowalske, Jeffrey C Schneider, Kristine A Parker, Elizabeth Flores

**Affiliations:** Keck School of Medicine, University of Southern California, Los Angeles, CA; Department of Medicine, Harbor-University of California Los Angeles Medical Center, Los Angeles, CA; Division of Plastic and Reconstructive Surgery, Keck School of Medicine, University of Southern California, Los Angeles, CA; University of Washington Department of Rehabilitation Medicine, Seattle, WA; Department of Physical Medicine and Rehabilitation, University of Texas Southwest Medical Center, Dallas, TX; Spaulding Rehabilitation Hospital, Mass General Brigham, Harvard Medical School, Boston, MA; University of Washington Medicine Regional Burn Center, Harborview Medical Center, University of Washington, Seattle, WA; Keck School of Medicine, University of Southern California, Los Angeles, CA

## Abstract

**Introduction:**

Burn injuries are functionally limiting, and pre-existing mobility impairments (PIMI) may further complicate recovery. PIMI is associated with worse outcomes in other populations; however, its role in burn recovery remains unclear. This study aims to evaluate the impact of PIMI on outcomes after burn injury.

**Methods:**

This retrospective cohort study was conducted from 2018 to 2022, utilizing data from adult participants in a national burn database. PIMI was defined as pre-burn physical problems or impairments affecting mobility (difficulty moving your arms, legs, or body). Outcomes included length of stay, discharge disposition, and PROMIS Global Mental Health scores at 6- and 12-months follow-up post injury. All regression models adjusted for age, total body surface area (TBSA), and inhalation injury. The model for Global Mental Health scores additionally adjusted for discharge location, Global Physical Health, pre-injury Global Mental Health, and follow-up time.

**Results:**

A total of 1372 adult burn survivors were included, with a mean age of 46.2 (Table 1). PIMI was associated with longer hospital stay (IRR 1.15, 95% CI [1.04 - 1.27], *p*=.006, Table 2) and discharge to shelter (OR 1.75, 95% CI [1.41- 2.19], *p*<.001, Table 2). There was also a trend toward lower PROMIS Mental Health scores among survivors with PIMI (β = –1.92, *p*=.091, Table 2).

**Conclusions:**

Pre-existing mobility impairment (PIMI) is associated with poorer burn outcomes, including longer hospital stay and discharge to unstable settings such as shelters, and may also be linked to poorer mental health outcomes. It remains unclear whether shelter discharge reflects premorbid housing instability or a new post-burn disposition.

**Applicability of Research to Practice:**

Patients with PIMI may benefit from targeted rehabilitation interventions in both acute and post-acute settings, which may reduce hospitalization and improve psychosocial outcomes.

**Funding for the study:**

The contents of this manuscript were developed under a grant from the National Institute on Disability, Independent Living, and Rehabilitation Research (NIDILRR grant numbers 90DPBU0007 and 90DPBU0008). NIDILRR is a Center within the Administration for Community Living (ACL), Department of Health and Human Services (HHS). The contents of this manuscript do not necessarily represent the policy of NIDILRR, ACL, or HHS, and you should not assume endorsement by the Federal Government.

